# On-chip dielectrophoretic single-cell manipulation

**DOI:** 10.1038/s41378-024-00750-0

**Published:** 2024-08-26

**Authors:** Zuyuan Tian, Xihua Wang, Jie Chen

**Affiliations:** 1 Department of Electrical and Computer Engineering, University of Alberta, Edmonton, AB T6G 1H9 Canada; 2 Academy for Engineering & Technology, Fudan University, Shanghai, 200433 China

**Keywords:** Electrical and electronic engineering, Physics

## Abstract

Bioanalysis at a single-cell level has yielded unparalleled insight into the heterogeneity of complex biological samples. Combined with Lab-on-a-Chip concepts, various simultaneous and high-frequency techniques and microfluidic platforms have led to the development of high-throughput platforms for single-cell analysis. Dielectrophoresis (DEP), an electrical approach based on the dielectric property of target cells, makes it possible to efficiently manipulate individual cells without labeling. This review focusses on the engineering designs of recent advanced microfluidic designs that utilize DEP techniques for multiple single-cell analyses. On-chip DEP is primarily effectuated by the induced dipole of dielectric particles, (i.e., cells) in a non-uniform electric field. In addition to simply capturing and releasing particles, DEP can also aid in more complex manipulations, such as rotation and moving along arbitrary predefined routes for numerous applications. Correspondingly, DEP electrodes can be designed with different patterns to achieve different geometric boundaries of the electric fields. Since many single-cell analyses require isolation and compartmentalization of individual cells, specific microstructures can also be incorporated into DEP devices. This article discusses common electrical and physical designs of single-cell DEP microfluidic devices as well as different categories of electrodes and microstructures. In addition, an up-to-date summary of achievements and challenges in current designs, together with prospects for future design direction, is provided.

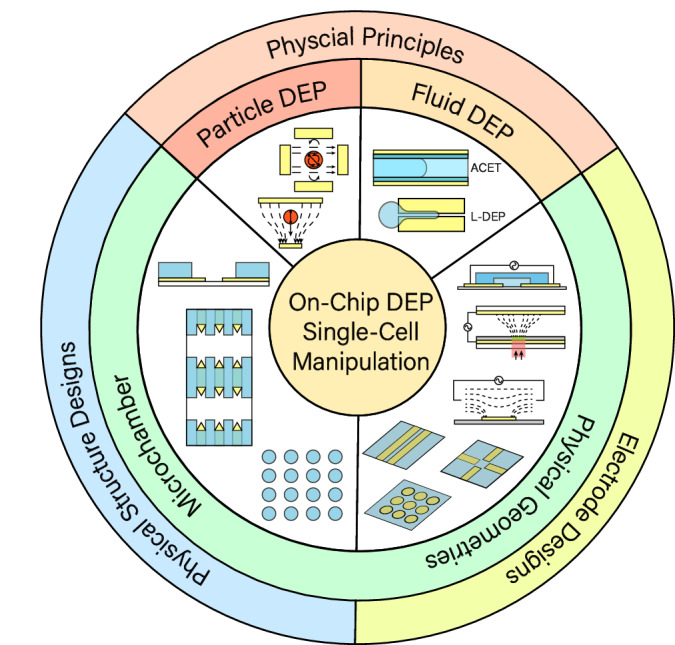

## Introduction

Conventional bioanalyses result in data from collective cell populations, thereby obscuring differences among individual cells. However, cell-to-cell variations can offer critical insights into underlying cellular heterogeneity and have provided us with unprecedented understanding of diverse biological processes, such as complex tumor ecosystems^1^, embryonic development^2,3^, and fluctuations in mammalian transcription events^4^. These significant discoveries have unequivocally demonstrated the significance of research at the single-cell level. Past decades have witnessed the development of numerous technologies that facilitate the study of large numbers of individual cells. Early single-cell analysis techniques relied on traditional methods such as microscopic imaging and patch-clump techniques. These techniques require manual separation and measurement approaches for single cells from suspension, thus yielding low throughput^5^. Automated fluorescent or laser scanning flow cytometry analyses have higher throughput but require significant benchtop space and only provide limited data points^6,7^. In recent years, microfluidic technologies have spawned the advancement of Lab-on-a-Chip technologies which aim to convert lab-scale analyses into chip-scale. Since microfluidic systems consist of microstructures comparable to cellular dimensions, miniaturized automatic high-throughput analysis of single cells has become possible.

Microfluidics allows for the manipulation of biological samples to assist in chemical or physical analysis via on-chip or off-chip^8^ methods. In order to support single-cell analysis, microfluidic technologies must be able to control the movement of a single cell across a chip. Methods addressing particle manipulation typically involve the application of specific microstructures that enable interference with the particle’s hydrodynamic states. Pressure-controlled flexible microvalves and micropumps, fabricated using multilayer elastomeric polydimethylsiloxane (PDMS) channels, have demonstrated utility in individual cell capture and forming boundaries for micro-reaction chambers^9,10^. Similarly, branched microchannels embedded with microtraps featuring variable geometry before and after cell trapping allow parallel single-cell capture^11^. In addition, specifically engineered micropillars can act as traps for single cells^12,13^ or filters to continuously disrupt microbial clusters for single-cell isolation^14^. The fluid shear rate coupled with wall interaction-induced repulsive force produces a hydrodynamic inertial effect that arranges the lateral particle position within low-Reynolds-number flows^15,16^. In single-cell analysis, inertial microfluidic devices with spiral or curved microchannels are capable of debulking the sample through a sheathless cell alignment, as shown in prior studies^17,18^. Although these hydrodynamic approaches have found applications in various fields, the passive realization of particle manipulation presents challenges in adjustability and programmability.

Incorporating multiple techniques into single-cell microfluidic devices to actively manipulate bioparticles has gained widespread interest in improving the capability of such devices. For instance, specifically labeling cells with immunomagnetic beads and subjecting the cells to a magnetic force via micromagnets can allow for efficient sorting and capture of target cells from complex samples^19^. Alternatively, label-free approaches such as surface acoustic wave (SAW) devices employ acoustic piezoelectric transducers to generate acoustic radiation force (ARF) and drag force caused by acoustic streaming flow (ASF)^20,21^. These acoustic-induced forces can be effectively harnessed for single-cell trapping and size-based selection^22,23^. Optical tweezers achieve active cell control with higher precision and programmability than magnetic and acoustic methods. Within microfluidic systems, lasers can either precisely control the movement of individual cells or trap a variety of cells in parallel using a microlens array^24,25^. Nevertheless, the laser-induced force, typically in the order of pico-Newtons, restricts cell capture to low flow rates, which significantly impacts the overall throughput. Additionally, the bulky peripheral instrumentation required for generating optical tweezers conflicts with the miniaturization objective of microfluidic systems.

DEP has emerged as a promising, label-free electrical technique for facilitating single-cell analysis due to its ability to address several limitations inherent in other methods mentioned above, including lacked programmability, adaptability, and low force amplitude. The fundamental principle of DEP involves inducing multipole momentum in polarized particles that interact with a non-uniform electric field, resulting in single-cell DEP force on the order of nanonewtons^26,27^. The motion of polarized particles in DEP mainly relies on the gradient of the electric field, rendering the technique highly adaptable through the application of varied DEP signals and electrode geometries. Since DEP is a pure electrical approach, advances in microfabrication technology enable the integration of DEP on the chip scale and have led to the creation of the architecture with programmable electrode arrays, allowing the arbitrary movement of multiple single cells, which currently remain unattainable in other methods^28^. Furthermore, the magnitude of DEP force depends not only on the particle’s geometry, but also on its dielectric properties. This added level of specificity enhances the applicability of DEP in comparison to other label-free approaches^29^.

**Fig. 1:**
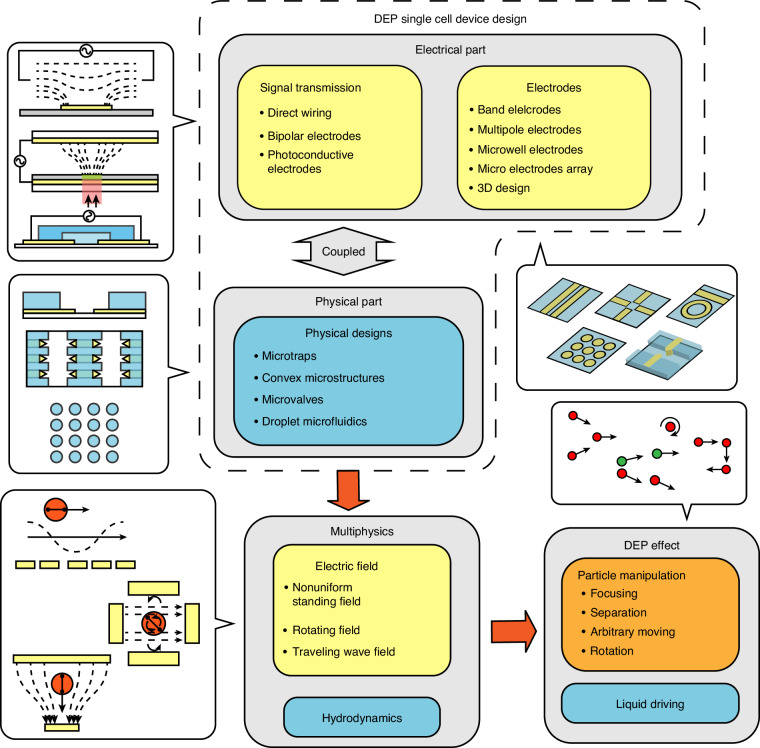
Diagram illustrating interrelations among electrical and physical designs of DEP designs, physics fields, and the resultant effects of DEP

As DEP-based microfluidic devices continue to gain prominence in single-cell analysis, this review primarily adopts an engineering perspective to provide design insights. The structure of this review begins with explanations of the governing physics, including different DEP effects and electrode driving methods. We then systemically categorized the state-of-the-art microelectrode and microstructure configurations of such devices based on geometry and functionality, briefly discussing their single-cell applications to offer a concise and comprehensive guide to interested scholars. A diagram illustrating the logical relationship between the introduced topics, including DEP device design, physics, and the DEP effect, is included (Fig. 1). The specific analysis principle and application scenarios of microfluidic single-cell bioanalysis, encompassing both DEP and non-DEP approaches, have been reviewed extensively and thus will not be reiterated here^8,30–32^. Finally, we summarize the role of DEP in contemporary microfluidic single-cell devices with existing challenges and provide our insights on future developments.

## Physical Principles

**Fig. 2:**
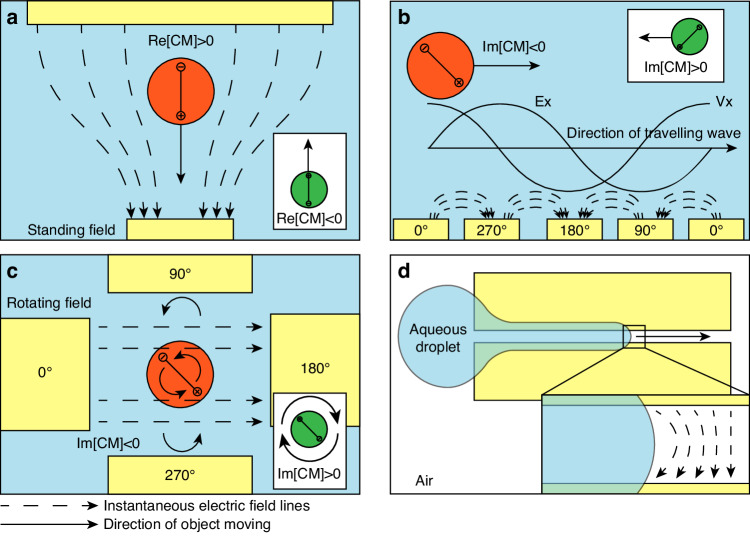
Schematic representations of different DEP effects. **a** DEP force in a standing electric field. The red particle represents the positive DEP force, and the green particle represents the scenario for the negative DEP force. **b** twDEP force and **c** ROT in a traveling-wave and a rotating electric field. The red particle represents the scenario when the phase lag of the induced dipole moment behind the electric field is smaller than $180^{\circ}$ (Im[CM] < 0), and the green particle represents the scenario when the phase lag is larger than $180^{\circ}$ (Im[CM] > 0). **d** Droplet movement induced by L-DEP force

DEP is attributed to the motion of dielectric objects in a non-uniform electric field. Under the DEP effect, dielectric materials exhibit induced dipole moments arising from the Maxwell-Wanger interfacial polarization, where differences in dielectric properties between the particle and the medium cause charge accumulation at the interface. The local electroneutrality assumption ensures insignificant electromigration, thereby maintaining stable interaction between the electric field and the induced dipole moment, generating DEP forces^33^. A distinct significant characteristic of DEP is its quadratic dependence on the applied electric field, as the induced dipole moment in the particles is generated by the external voltage itself. The quadratic relation reveals its nondirectional dependence on the electric field. Therefore, DEP can be effectively utilized under the actuation of alternating current (AC) voltage signals, which is also advantageous over the direct current (DC) voltage due to the reduced electrolysis and heating^34^. For particle manipulation, the DEP effect can be categorized into DEP in the standing wave electric field and DEP in the dynamic wave electric field, depending on the applied electric field. The standing wave electric field with uniform phase distribution has spatially invariant and time-fixed extremum regions, which are only influenced by the geometry boundaries. DEP in the dynamic wave field mainly relies on the time-dependent dynamics of the electric field induced by the spatially non-uniform phase distribution. Consequently, DEP in the dynamic electric field can result in more complex motions, such as electrorotation (ROT) in a rotating field or long-distance particle transport in a traveling wave electric field (twDEP)^35–37^ as shown in Fig. 2. Also, DEP force can be exerted on dielectric liquid droplets at the liquid-gas interface, promoting liquid dielectrophoresis (L-DEP)^38^. Additionally, within a one-phase non-isothermal liquid buffer, the gradient of dielectric properties can arise due to the thermal-dependent ion mobility. DEP force resulting from this thermal-induced conductivity and permittivity gradient can also induce the fluid flow, known as the AC electrothermal (ACET) flow.

### Standing Wave DEP

DEP force acting on a particle can be modeled as a dipole moment experiencing electric force in a non-uniform electric field. In a standing wave sinusoidal electric field with uniform phase distribution, where the phase of $\bar{{\bf{E}}}{\boldsymbol{(}}{\bf{r}}{\boldsymbol{)}}$ is not dependent on ${\bf{r}}$, the expressing of time-averaged standing wave DEP force is given by^39^:

1 $$\begin{array}{c}\left\langle {{\bf{F}}}_{{\rm{DEP}}}({\bf{r}})\right\rangle = {\rm{\pi }}{{\rm{\varepsilon }}}_{m}{{\rm{r}}}^{3}\mathrm{Re}\left(\frac{\bar{{{\rm{\varepsilon }}}_{p}}-\bar{{{\rm{\varepsilon }}}_{m}}}{\bar{{{\rm{\varepsilon }}}_{p}}+2\bar{{{\rm{\varepsilon }}}_{m}}}\right){\boldsymbol{\nabla }}{\left|\bar{{\bf{E}}}({\bf{r}})\right|}^{2} = {\rm{\pi }}{{\rm{\varepsilon }}}_{m}{{\rm{r}}}^{3}\mathrm{Re}[{\rm{CM}}\left({\rm{\omega }}\right)]{\boldsymbol{\nabla }}{\left|\bar{{\bf{E}}}({\bf{r}})\right|}^{2}\end{array}$$

Assuming constant conductivities and permittivities of the material, the bracketed fraction in Eq. (1) solely depends on angular frequency, mathematically characterizing the relative polarization intensity between the particle and medium, and is termed the Clausius-Mossotti (CM) factor. A positive CM factor indicates that particles are more polarizable than the medium, resulting in DEP force with the direction along the electric field gradient, also known as positive DEP (pDEP) (Fig. 2a). Conversely, particles less polarizable than the surrounding medium experience negative DEP (nDEP) which opposes the electric field gradient. For a single cell within a specific medium, the CM factor is only a function of frequency. The x-interception of this function corresponds to the crossover frequency, at which point the particle and the medium exhibit the same polarization intensity, and DEP force equals zero^40^. Numerous research has utilized the crossover frequency to study the single-cell dielectric spectra^41–43^. Moreover, cells of different types exhibit varying CM factors within the same medium, leading to DEP forces that point to different directions at the selected frequency. This property can be exploited to separate target cells from a mixed sample, which has given rise to DEP-assisted cell sorting (DACS) technology^44,45^.

### Dynamic Wave DEP

#### Traveling Wave DEP (twDEP)

In a standing wave field, a DEP force either repels or attracts particles to the field maxima, which is predetermined by electrodes, microstructure configuration, or a combination of both. This can effectively facilitate particle capture and release. However, due to the fixed field extremum points and the exponential decay of an electric field away from coplanar electrodes, the long-distance transport of particles in microfluidic chips using DEP force in a standing field presents challenges^46,47^. The basic concept of twDEP is to create a dynamic electric field with a series of high-speed traveling extremum points. This generates continuous interaction between the induced dipole and the field, providing a long-distance driving force for particles^48^. The dynamic electric field with traveling extreme points can be produced by sequentially arranging electrodes and applying phase-shifted signals (Fig. 2b). Incorporating the spatially varying phase distribution of the traveling wave electric field, where ${\bf{E}}\left({\bf{r}}\right)=\mathrm{Re}[\left|\bar{{\bf{E}}}{\boldsymbol{(}}{\bf{r}}{\boldsymbol{)}}\right|{e}^{{\rm{j}}{\rm{\varphi }}({\bf{r}})}]$, the additional term accounting twDEP effect in more comprehensive DEP expression is^49^:

2 $$\begin{array}{c}\left\langle {{\bf{F}}}_{{\rm{twDEP}}}\left({\bf{r}}\right)\right\rangle =2{\rm{\pi }}{{\rm{\varepsilon }}}_{{\rm{p}}}{{\rm{\varepsilon }}}_{0}{{\rm{r}}}^{3}{\rm{Im}}\left[{\rm{CM}}\left({\rm{\omega }}\right)\right]\left({\boldsymbol{\nabla }}{{\times }}\mathrm{Re}\left(\bar{{\bf{E}}}{\boldsymbol{(}}{\bf{r}}{\boldsymbol{)}}\right){{\times }}{\rm{Im}}\left(\bar{{\bf{E}}}{\boldsymbol{(}}{\bf{r}}{\boldsymbol{)}}\right)\right)\end{array}$$

Distinct from DEP in the standing field, twDEP is related to the out-of-phase (imaginary) part of the CM factor. Depending on whether the positive or negative of ${\rm{Im}}\left[{\rm{CM}}\left({\rm{\omega }}\right)\right]$, the translational force from twDEP effect will direct the particle along with or reverse the direction of electric field proporgation^50^.

#### Electrotation (ROT)

A non-uniform phase distribution of the electric field can also induce the rotating electric field by arranging signal sources with linearly increasing phases to form a closed circle. It is noteworthy to mention that a phase lag exists between the induced dipole moment and electric field due to the existence of the CM factor, implying that this lag arises from different polarization intensities between the medium and particle. In a rotating electric field, the rotation of the induced dipole moment will also lag behind the rotation of the electric field. This lag results in the angle between the induced dipole moment and the electric field, leading to the rotation of polarized particles (Fig. 2c). The time-averaged induced torque in a circular rotating field is calculated by^35^:

3 $$\begin{array}{c}\left\langle {{\mathbf{\Gamma }}}_{{\rm{DEP}}}\left({\bf{r}}\right)\right\rangle ={\boldsymbol{-}}4{\rm{\pi }}{{\rm{\varepsilon }}}_{m}{{\rm{r}}}^{3}{\rm{Im}}\left[{\rm{CM}}\left({\rm{\omega }}\right)\right]{\left|\bar{{\bf{E}}}\left({\bf{r}}\right)\right|}^{2}\hat{{\bf{z}}}\end{array}$$

where $\hat{{\bf{z}}}$ is the unit vector normal to the rotating surface. Combined with the resistive viscous torque, the rotation rate of a spherical dielectric particle in the medium of viscosity ${\rm{\eta }}$ is given by^51^:

4 $$\begin{array}{c}{\Omega }_{{ROT}}\left({\bf{r}}\right)=\frac{{{\rm{\varepsilon }}}_{m}{\left|\bar{{\bf{E}}}\left({\bf{r}}\right)\right|}^{2}}{2{\rm{\eta }}}{\rm{Im}}\left[{\rm{CM}}\left({\rm{\omega }}\right)\right]{\rm{\#}}\end{array}$$

Measuring the rotation rate of the ROT, therefore, provides another means of determining the dielectric property of individual cells^52^. Note that an accurate calculation of DEP, including ROT and twDEP, on single cells requires consideration of additional factors, including multipolar moments induced by the high-order electric field gradient^53^, the non-spherical geometry of cells^54,55^, and the multilayer structure of cells^56–58^.

### Fluid DEP

#### Liquid-DEP (Dielectroweting)

In addition to solid particles, the phenomenon of DEP can also be observed in dielectric fluids. In such cases, the liquid tends to accumulate in regions of high electric field intensity (Fig. 2d), while gas or vapor bubbles are repelled from strong fields^38^. The electrical force density acting on the liquid can be obtained from the Korteweg-Helmholtz equation:

5 $$\displaystyle{\begin{array}{c}{{\bf{f}}}_{{\bf{K}}{\bf{H}}}^{{\bf{e}}} = {{\rm{\rho }}}_{{\rm{f}}}{\bf{E}} - \frac{1}{2}{{\bf{E}}}^{2}{\boldsymbol{\nabla }}{{\rm{\varepsilon }}}_{m}+{\boldsymbol{\nabla }}\left(\frac{1}{2}{{\bf{E}}}^{{\boldsymbol{2}}}{{\rm{\rho }}}_{m}\frac{\partial {{\rm{\varepsilon }}}_{m}}{\partial {{\rm{\rho }}}_{m}}\right)\end{array}}$$

Here, ${{\rm{\rho }}}_{{\rm{f}}}$ represents the free electric charge density in the liquid, and ${{\rm{\rho }}}_{m}$ denotes the fluid density. The first term in Eq. (5) corresponds to the Coulombic force, and the second term signifies the DEP force due to charge dipoles. The third term, representing electrostriction, can be neglected for incompressible fluids. Coupled with the Navier-Stoke equations, the Maxwell stress tensor ${{\bf{T}}}_{{\rm{KH}}}$ derived from Eq. (5) mathematically characterizes the behavior of incompressible fluids subject to an electric field:

6 $$\begin{array}{c}{\boldsymbol{\nabla }}\,\cdot \,{{\bf{T}}}_{{\rm{KH}}}={{\bf{f}}}_{{\bf{K}}{\bf{H}}}^{{\bf{e}}}={\boldsymbol{\nabla }}\,\cdot\, ({{\rm{\varepsilon }}}_{m}{\bf{E}}\otimes {\bf{E}}-\displaystyle\frac{{\bf{1}}}{{\bf{2}}}{{\rm{\varepsilon }}}_{m}{{\bf{E}}}^{{\bf{2}}}{\bf{I}})\\ {{\bf{T}}}_{{\rm{KH}}}={{\rm{\varepsilon }}}_{m}{\bf{E}}\otimes {\bf{E}}-\frac{{\bf{1}}}{{\bf{2}}}{{\rm{\varepsilon }}}_{m}{{\bf{E}}}^{{\bf{2}}}{\bf{I}}\end{array}$$

where ${\bf{I}}$ is the unit tensor. The force density ${{\bf{f}}}_{{\bf{K}}{\bf{H}}}^{{\bf{e}}}$ in Eq. (6) can be expressed as the divergence of ${{\bf{T}}}_{{\rm{KH}}}$, neglecting the compressible term. The overall force on the fluid body can be calculated in bulk form to integrate the ${{\bf{f}}}_{{\bf{K}}{\bf{H}}}^{{\bf{e}}}$ through the liquid volume or the surface integral along the bounding of liquid with ${{\bf{T}}}_{{\rm{KH}}}$. L-DEP, functioning as an electrically driven, pump-free approach to actuate fluid movement, can be effectively utilized for the formation and manipulation of liquid droplets as well as the enhancement of liquid sample mixing^59^. One of the prevalent applications of Liquid-DEP (L-DEP) in microfluidic devices is the manipulation of dielectric droplets. In this case, L-DEP, also referred to as dielectrowetting (DEW), induces an unsymmetric alternation of the contact angle of low-conductivity dielectric droplets. This generates an interfacial tension gradient, allowing controlled displacement of dielectric droplets. Electrowetting on dielectric technology (EWOD), which can transport conductive droplets, integrated with conductive and dielectric droplet manipulation forms a digital microfluidic platform^60^. Numerous studies have demonstrated the capacity of digital microfluidic devices for signal-cell analysis^61,62^.

#### AC Electrothermal Flow

DEP force can also drive the flow of one-phase non-isothermal fluid. This phenomenon differs from the particle or droplet DEP force, which applies to two-phase substances, where the different polarization intensities arise from intrinsic differences in the dielectric properties of two materials. To drive one-phase flow with DEP force, the change or the gradient of the dielectric properties within the fluid is established along with the thermal gradient, so the corresponding DEP-driven flow also refers to the AC electrothermal flow (ACET). The calculation of the force density of ACET flow involves the electrical force density described by Eq. (6) and the temperature field described by the energy balance equation related to the temperature field^63^, which are coupled by the relationship between the temperature and the dielectric properties of the fluid. Under assumptions of weak temperature gradient and neglecting the change in liquid properties with respect to the temperature, the time-averaged force density of the electrothermal flow can be written as^64^:

7 $$\begin{array}{c}\left\langle {{\bf{f}}}_{{\bf{ET}}}\right\rangle =\frac{1}{2}\mathrm{Re}\left[\frac{{{\rm{\varepsilon }}}_{m}^{0}\left({{\rm{T}}}_{0}\right)\left({\rm{\alpha }}-{{\beta }}\right)}{1+{\rm{j}}{{\omega }}{{\tau }}}\left({\boldsymbol{\nabla }}{\rm{T}}\,\cdot\, {{\bf{E}}}_{0}\right){{\bf{E}}}_{0}^{* }\right]-\displaystyle\frac{{{{\varepsilon }}}_{m}^{0}}{4}{\rm{\alpha }}({{\bf{E}}}_{0}\,\cdot\, {{\bf{E}}}_{0}^{* }){\boldsymbol{\nabla }}{\rm{T}}\end{array}$$

where the permittivity and the conductivity change linearly with the temperature, each given by ${{{\varepsilon }}}_{m}\left({\rm{T}}\right)={{{\varepsilon }}}_{m}^{0}\left(1+{{\alpha }}\left({\rm{T}}{{\mbox{-}}}{{\rm{T}}}_{0}\right)\right)$ and ${\sigma }_{m}\left({\rm{T}}\right)={\sigma }_{m}^{0}\left(1+{{\beta }}\left({\rm{T}}{{\mbox{-}}}{{\rm{T}}}_{0}\right)\right)$, respectively. Here, ${{\rm{\varepsilon }}}_{m}^{0}$, ${\sigma }_{m}^{0}$, ${\rm{\alpha }}$ and ${\rm{\beta }}$ are the quantities and their relative derivatives at ${\rm{T}}={{\rm{T}}}_{0}$. ${{\bf{E}}}_{{\bf{0}}}$ represents the electric field for a spatially constant temperature at ${\rm{T}}={{\rm{T}}}_{0}$. ${\rm{\tau }}$ is the induced charge relaxation time characterized by the conductivity and the permittivity, ${\rm{\tau }}={{\rm{\varepsilon }}}_{m}^{0}/{\sigma }_{m}^{0}$. More enhanced models have also been proposed to calculate the electrothermal force denstity^65^.

Similar to the particle DEP, ACET can operate in both standing and traveling wave electric fields, referring to standing wave electrothermal flow (SWET) and traveling wave electrothermal flow (TWET)^66^. In a microfluidic device, the ACET can be simply generated with coplanar electrodes with the internal joul heating or external heat source at the top or bottom that induces the vertical temperature gradient. In this case, the SWET effect is induced by the in-phase polarization, producing vortex flow pairs above electrodes. The flow direction can be controlled by the frequency of the electric field being smaller or larger than the cross-over frequency, determining whether the domination force is either Coulomb force or dielectric force^67^. By contrast, the TWET flow is induced by the out-of-phase polarization in an electric field with periodic phase distribution, capable of driving the streaming flow along or reversing the direction of the field proporgation^66^. Additionally, with the electrode configuration generating a rotating electric field, ACET can also produce the horizontal vortex that rotates the aqueous solution^64^. The detailed principles and physics of the ACET have been studied and extensively reviewed^63,65,68^.

## DEP signal transmitting methods

**Fig. 3:**
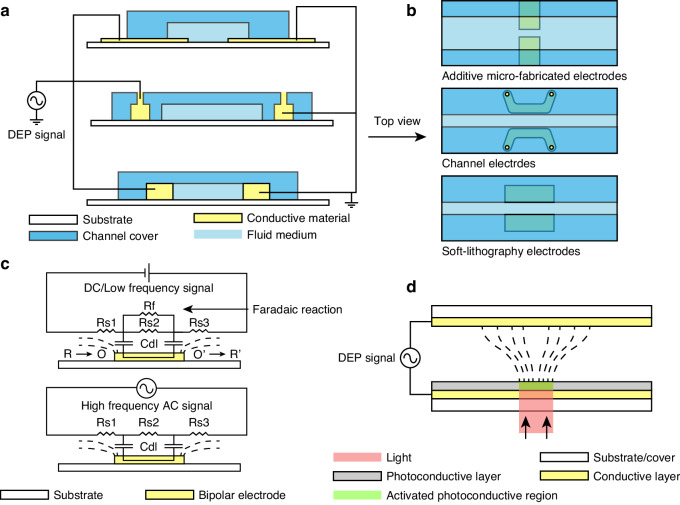
Schematic of different DEP signal transmitting methods. **a**, **b** Directing wiring approach with deposited microelectrodes, channel electrodes, and soft-lithography electrodes. **c** Wireless signal with bipolar electrodes under low-frequency and high-frequency electric fields and corresponding equivalent circuits. Rs refers to the solution resistance, Cdl refers to the electrical double layer capacitance, and Rf refers to the resistance to faradaic reactions. **d** Definition of electrode shapes by optical pattern projection with photoconductive electrodes

Generating non-uniform electric fields for microfluidic systems demands the establishment of electrical connections between signal sources and on-chip electrodes. This section provides an overview of the approaches for transmitting DEP signals to fluid channels in single-cell microfluidic chips. Categorized based on their physical principles, these signal transmission approaches include: direct electrical wiring, bipolar electrodes, and photoconductive electrodes, as outlined in Fig. 3.

### Direct electrical wiring

Direct connection of on-chip electrodes to the signal source is the most common method for DEP signal transmission. Leveraging micro/nano-fabrication and electrical chip packaging techniques, establishing metal connections between electrodes and the signal routing pads can be readily achieved. Like integrated circuits (ICs), this type of electrical wiring is defined on the device substrate through semiconductor processes, such as deposition and etching^69^. Aside from direct additive fabrication, microfluidic systems can also form the electrodes and wires by filling channels with conductive materials, such as high-concentration saline buffer and molten solder^70,71^. As these channel-formed electrodes do not have direct contact with sample flow channels, the DEP effect generated by this type of electrode is referred to as contactless DEP (cDEP)^72^. Soft-lithography technology, a standard method for fabricating fluid channel covers, can also be employed for electrode fabrication using conductive silicone composites, such as silver-polydimethylsiloxane (AgPDMS)^73^. Due to different fabrication processes, these electrodes are typically constructed in conjunction with the channel cover, rather than on the substrate or sandwiched between the substrate and channel cover (Fig. 3a, b).

### Wireless bipolar electrode

Bipolar electrodes (BPEs) arefloating electrodes in a liquid solution. BPEs are activated by applying a voltage to a pair of driving electrodes situated on two sides of the device, generating an approximate linear potential gradient through the solution above the BPEs^74^. Faradaic reactions at the BPE/electrode interface occur when the potential drop at two ends of BPE is sufficient to drive both reduction and oxidation reactions of available redox species in the medium^75^. The exchange current density enhances electric field leakage perpendicular to the surface, creating a strong electric field region in the ion-depleted zone. This bipolar faradic concentration polarization directly affects the electric field gradient above BPE. DEP force is typically generated under an AC electric field with a frequency higher than the rate of electron transfer for available redox species. In this case, the main current route becomes charging and discharging of the electrode double layer (EDL) of BPEs that shares the current form solution, resulting in the sudden change of potential at two ends of the electrodes without faradaic reactions. Maximum electric fields occur at the edges of the BPEs where the largest potential change exists, while minimum field intensity occurs at the center of the floating electrodes as the electrode is an equipotential object^76^. These local electric field peaks and troughs facilitate on-chip DEP with wireless electric field transmission (Fig. 3c). Compared to direct electrical wiring, this wireless approach allows simultaneous activation of a microelectrode array (MEA) with a monolayer metal deposition. Wireless transmission demands a high driving voltage, ranging from tens to over a hundred volts, as opposed to several to tens of volts required for direct wiring. Under a DC or low-frequency field, high voltage can stimulate electrochemical reactions, causing faradic ion enrichment (FIE) and faradic ion depletion (FID) at two ends of the BPEs. These phenomena have been exploited to shape and extend the electric field gradients responsible for DEP force^77^.

### Photoconductive electrode

Conventional electrode configurations remain fixed once fabricated, demanding complex microelectronic design and advanced CMOS fabrication processes to achieve high-resolution, arbitrary manipulation of particles with DEP force^28^. Photoconductive materials, such as hydrogenated amorphous silicon (a-Si:H), permit an alternative approach for programmable manipulation. These materials exhibit high resistivity in the absence of light. While upon light illumination, their resistance decreases by many orders of magnitude due to the generation of electron-hole pairs^78^. By depositing thin-film photoconductive material on a transparent conductive layer, such as indium-tin-oxide (ITO), the shape of virtual electrodes determining the electric field distribution can be defined and readily altered with custom light projections^79^. This method, known as optoelectronic tweezers (OET) or optical DEP (ODEP), provides a dynamic and versatile strategy for DEP-based microfluidic particle manipulation (Fig. 3d). In comparison to purely optical-driven approaches like the optical tweezer, OET can generate a larger force by involving the DEP effect. Also, OET requires relatively less bulky and less costly instrumentation for optical pattern projection in contrast to high-power laser beam generation required by optical tweezer systems^80^.

## Electrode design

The determination of the electric field that can generate the desired single-cell motion through DEP force involves considering both the physical geometry and applied signal. Based on the geometry, electrode patterns are classified into four categories: band electrodes, microwell electrodes, quadrupole electrodes, and microelectrode arrays. An additional sub-section will discuss 3-D electrodes, which can be viewed as a vertical stretch of the above-listed 2-D electrode patterns.

### Cascaded band electrodes

By placing a pair of planar rectangular band electrodes either adjacent to or opposite to each other and applying a potential difference between them, a non-uniform electric field is generated within the space. This configuration yields the simplest method to create an on-chip DEP. As electrodes are equipotential surfaces and the electric field intensifies at sharp edges, the maximum and minimum field gradients occur at the edges and middle sections of the electrodes, respectively. This results in the formation of a line-shaped region where the electrical field reaches the extremums.

For different application purposes, the shape and orientation of these line-shaped extremum regions can be modified to change the spatial distribution of the gradient extremums. With standing DEP, such configurations are suitable for inducing short-distance particle displacement or confining the direction of particle movement, such as cell trapping and focusing in microchannels. For dynamic DEP, cascaded rectangular band electrodes can generate the traveling wave electric field by applying a step phase increase, typically +90°, to each adjacent electrode. The direction of the traveling wave corresponds to the direction of phase increase. Additionally, electrode pairs form the most commonly employed configuration to generate the L-DEP and ACET flow.

#### Orientation and shape of band electrode pairs

Electrode pairs can be arranged in various orientations to control or influence the movement of particles by coupling DEP force and flow drag force. Through the planar fabrication process, electrodes can be designed to cover the channel’s bottom/top to influence particles from the vertical side or be positioned along channel sidewalls to influence particles from the lateral side. Based on the angle between the central axis of electrode pairs and channels, parallel electrode pairs at the channel bottom/top can be categorized into three sets: ${\rm{\theta }}=90^{\circ}$, $0^{\circ} \,<\, {\rm{\theta }}\, <\, 90^{\circ}$, and ${\rm{\theta }}=90^{\circ}$.

When electrode pairs are arranged perpendicular to the flow direction, horizontal components of DEP force will act in an exact opposing direction to the Stokes’ drag force if particles flow through electrodes. Therefore, DEP force can function as a physical barrier to trap or impede the movement of cells, facilitating the following on-chip optical characterization^81^. The interdigitated electrode (IDE) has been employed to concentrate cells with pDEP force in a microfluidic device^82,83^. When cells are captured by IDEs, their DEP force is balanced with the drag force. Given a known geometry and flow condition, it is possible to calculate the CM factor and dielectric properties of cells^84^. In single-cell applications, electrodes can also be modified into zigzag or jagged shapes to generate gradient extremums in a more confined range to capture single cells^85^. pDEP attracts cells to electrodes where DEP force strengthens, while nDEP force repels cells towards the region with weaker DEP force. Hence, cells undergoing pDEP are easier to trap than cells undergoing nDEP. Microfluidic systems leverage perpendicular arranged IDEs to selectively sort cells undergoing pDEP force from particles undergoing nDEP^86,87^. As the high gradient appears at the electrode edges, the IDE can be modified to a castellated shape to have a larger edge area^88^ to facilitate selective capture with pDEP. These cell sorting designs operate in a non-continuous format, where cells exhibiting a pDEP response are selectively separated from other cell types. Other stream-based cell sorting designs operate continuously, utilizing the vertical components of DEP force to bring cells to different fluid layers. Several proposed DEP/G-FFF (gravitational field-flow fraction) devices utilize an nDEP force coupled with the gravitational force to level various cell types into different heights in the channel. The parabolic flow profile amplifies this small vertical height difference, on the order of tens of microns, to a substantial horizontal position change, inducing a several-minute arrival time difference at the outlet port^89,90^. An alternative stream-based strategy determines the vertical position of particles based on the nDEP and pDEP states of different cells by employing a COMS microfluidic cytometer design. The system differentially measures the signal change of particles as they pass by two-spaced sensing electrodes. Since the signal amplitude is directly related to the height of particles in the channel, an increase or decrease in signal amplitude corresponds to the influence from nDEP or pDEP, enabling high-throughput electrical single-cell DEP analysis^91^. For such top or bottom electrode design, to ensure that all cells are within the effective range of DEP, the channel height must be sustained to a specific range, which also constitutes the key limitation for device throughput.

When the electrode pairs are oriented at an acute angle to the fluid flow, the DEP force will hinder the movement of cells perpendicular to the electrodes, and cells will have a tendency to move along the electrode pairs. If the DEP force is sufficiently strong, electrodes can serve as a virtual track and laterally transport cells along electrodes in the channel. Under such conditions, a small variation in the DEP force will only influence the position of cells relative to electrode edges. Measuring such position change at different frequencies enables microfluidic-based rapid single-cell DEP characterization^92^. This virtual track can also be used to change the lateral position of cells in the channel, such as concentrating cells into a thin streamline. It offers a sheathless and high throughput approach for microfluidic particle focusing^93^. Also, DEP force is proportional to the volume of the particle, while the fluid drag force is proportional to the radius of the particle. As particle size shrinks, the effect of the DEP force will become less dominant compared to the drag force, allowing immunocyte discrimination based on size and DEP phenotype^94^. Cell sorting with tilted electrode pairs can also be accomplished based on different DEP responses, considering the nDEP effect is typically weaker than pDEP with the same signal. When particles undergoing pDEP pass the tilted IDE, cell attachment is a common issue that arises due to the strong pDEP. Solutions to this challenge include using a thin passive layer and modulating the DEP signal to alternate between on and off states at several hertz^93,95^.

When the electrode pair is positioned parallel to the flow direction, the horizontal components of the DEP force act perpendicularly to the drag force. A common application scenario of such designs is stream-based cell sorting devices, which separate cells based on the state of DEP response. In these devices, the electrode shape is usually modified to a non-straight shape to amplify the lateral distance between the maximum and minimum of the gradient field and direct cells to different channel branches^96^. With the appropriate buffer and DEP signal allowing cells to exhibit the same type of DEP response, parallel electrode configuration can also be utilized to focus cells for subsequent single-cell characterization, such as impedimetric flow cytometry^97^. In comparison to the tilted electrode pairs that can generate a stable lateral DEP force across the channel width, the parallel design is less suitable for inducing a long lateral translation at a high flow rate. In addition to particle DEP force, ACET vortices perpendicular to the flow direction, induced by this parallel electrode configuration, have been employed to facilitate the in-droplet cell/synthetic particle concentration^98^.

Another method to generate an electric field gradient in a lateral direction is to arrange the electrodes at the side of the channel. Similar to the perpendicular electrode at the top or bottom of the channel that directs cells to different channel heights, the DEP force generated by electrodes positioned at the side of the channel directs cells to different lateral positions. One advantage of the side electrode design is that lateral channel branches are easier to fabricate than vertical branches. Nevertheless, to ensure that the electric field influences all particles passing through electrode pairs, side electrode designs are typically used with narrow channels^99–101^. A trapezoidal electrode design provides an alternative strategy to extend the electrode from the channel side to cover the whole channel bottom while maintaining the lateral gradient^102^. However, an increase in lateral DEP influential range was not observed in this design. A systemic comparison and summary of the aforementioned orientations and arrangements of electrode pairs, including their designs, applications, and the constrained channel dimensions to ensure all cells are within the effective range, are provided in Table 1.

**Table 1:** Comparison of different arrangements and orientations of band electrode pairs

Arrangements and Orientations	Electrode Designs	Applications	Channel Dimensions Constrained by DEP
	Straight	Cell capture^81^ Dielectric characterization^84^ Non-stream cell sorting^86^ Stream-based cell sorting^89,202^	Channel height
	Zig-zag	Cell capture^203^	
	Castellated	Cell capture^88^ Non-stream cell sorting^88^	
	Straight	Dielectric characterization^92^ Stream-based cell sorting^94^ Cell focusing^93^	Channel height
	Straight	Steram-based cell sorting^204^ Cell focusing^204,205^	Channel height and channel width
	Expanding gap	Stream-based cell sorting^96^	
	Narrowing gap	Cell focusing^97^ Stream-based cell sorting^76,206^	
	Straight	Stream-based cell sorting^99,207^	Channel height and channel width
	Trapezoidal	Stream-based cell sorting^102^	

Fluid Drag Force  Low Gradient Region

Horizontal Component of DEP Force  High Gradient region

Vertical Component of DEP Force (Outward/Inward to the Plane)

#### Traveling wave field generation

**Fig. 4:**
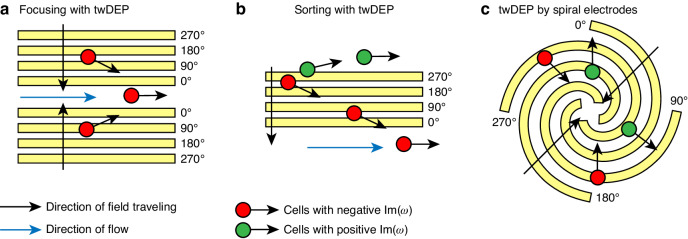
Operating schematics of twDEP generated by cascaded band electrodes. **a** Cell focusing with twDEP^104^. **b** Streamed-based cell sorting with twDEP^105^. **c** Non-stream-based cell sorting with twDEP generated by spiral electrodes^106^

Cascaded band electrodes represent the most common configuration for generating traveling wave electric fields by applying each adjacent electrode with a periodic phase-increasing signal^103^. Since the extremums of the gradient field also travel with the field, the magnitude of lateral twDEP force can be maintained through electrodes fabricated at the channel bottom, which makes this technique suitable for long-distance particle transfer. A key distinction from electrode designs operating in a standing electric field coupled with fluid drag force is that twDEP can manipulate cells in a static fluid environment. Microfluidic devices have utilized twDEP for lateral cell sorting and focusing in wide channels^104,105^ (Fig. 4a, b). The parallel band electrodes represent the most basic schematic for twDEP generation, where the electric field travels in one dimension. Multi-direction particle manipulation can be achieved with curved or spiral electrode designs (Fig. 4c)^106,107^.

#### Droplet driving

**Fig. 5:**
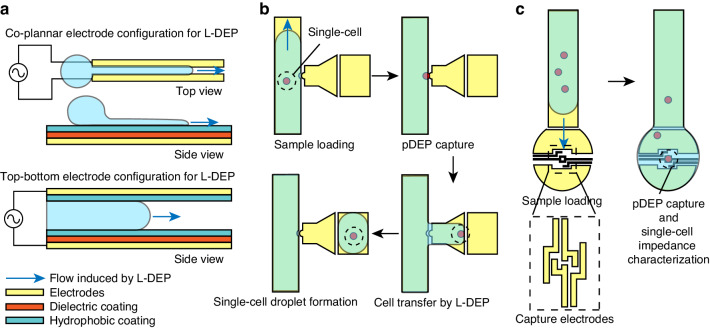
Structures of L-DEP device and its applications in single-cell analysis. **a** Co-planar and top-bottom electrodes to translate droplets with L-DEP. **b** Single-cell droplet formation by the coupling of L-DEP and pDEP^109^. **c** L-DEP device for pDEP single-cell capture and impedance characterization^112^

The fabrication process for L-DEP devices follows a similar approach to other digital microfluidic platforms. Typically, a dielectric layer fabricated by silicon nitride deposition or SU8 photoresist coating is deposited on the electrode surface to prevent potential electrolysis and Joule heating^59^. Additionally, a hydrophobic layer, such as Teflon, is coated at the liquid contact surface to allow the control of the horizontal fluid shape by L-DEP. In L-DEP devices, two poles of electrodes can be either fabricated on the top and bottom surfaces of the fluid channel or fabricated as coplanar electrode configurations^108^ (Fig. 5a).

Most digital microfluidic devices for single-cell analysis primarily rely on electrowetting, as bioanalysis buffers typically own high conductivity^109,110^. Therefore, applications of L-DEP or dielectrowetting in cell analysis are limited to scenarios where a low-conductivity buffer is utilized. For instance, microfluidic devices that integrate pDEP trapping can utilize the L-DEP technique to achieve pump-free fluid manipulation. In single-cell analysis, L-DEP has been employed to create a droplet environment for a single cell immobilized by pDEP^111^ (Fig. 5b). The same research group has also introduced another parallel plate microfluidic device employing L-DEP to transport cells to the target pDEP capture electrodes for single-cell impedimetric characterization^112^ (Fig. 5c).

### Quadru(multi)pole electrodes

Multiple electrodes typically consist of four (quadrupole), six (hexapole), or eight (octople) electrode poles arranged in circular or rectangular patterns. By applying the same electrical potential to each opposing electrode pole and a different electrical potential to adjacent electrode poles, the minimum of the electric field gradient will appear at the center of the multipole electrodes. Therefore, with nDEP force, such electrode configurations can be used to trap cells in a concentrated region. Besides, multipole electrodes can generate a rotating field by applying oscillating sources with linearly increased phases in a period.

#### DEP cage

**Fig. 6:**
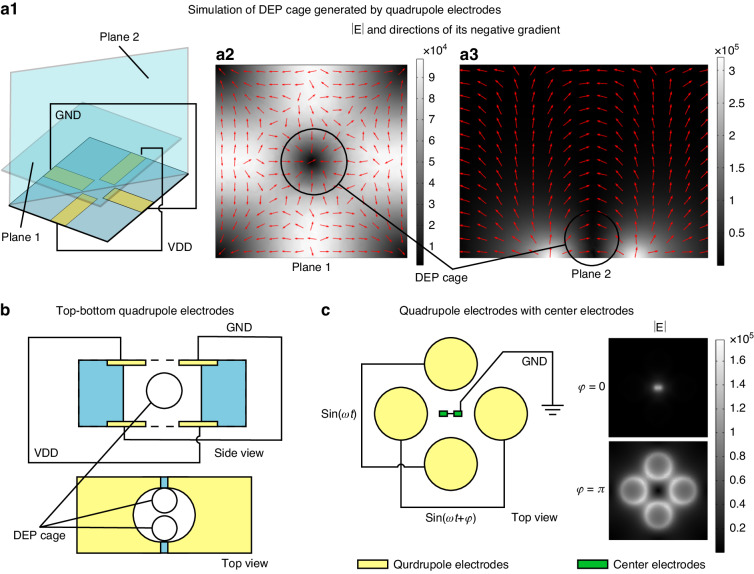
DEP cage generated by quadrupole electrodes. **a1** Schematic of quadrupole electrodes for DEP cage generation. Simulations of the electric field in the horizontal (**a2**) and vertical (**a3**) plane. **b** DEP cage generated by top-bottom quadrupole electrodes^117^. **c** Schematic and electric field simulations of quadrupole electrodes with center electrodes^118^

The term DEP cage has been used in many studies to describe the virtual electric field gradient minimum region that can trap cells with nDEP force. With a quadrupole electrode configuration, the DEP cage will be generated at the center by connecting the positive pole of the DEP signal source to one pair of opposing electrodes and ground to the other (Figure 6a1). The generated electric field distribution over the horizontal and vertical planes was simulated using the COMSOL finite element simulation tool, where a clear minimum region of the electric field norm representing the DEP cage was visualized (Figure 6a2, a3). It is notable that peaks of the electric field appear at the edge of electrodes, so multipole electrodes can also generate a pDEP force in a low-conductivity medium environment^113^.

In the horizontal plane, the DEP cage formed by the coplanar quadrupole electrode is clearly located at the center of the electrode pattern. By contrast, in the vertical plane, the minimum of the electric field norm is only half-surrounded by the high electric field region, which means cells at the center of the DEP cage are not trapped in the horizontal direction. With nDEP, cells are repelled from the electrodes and tend to be pushed to the top of the channel. Therefore, in designs that utilize coplanar multipole electrodes to generate DEP cages, channel height will become a significant design factor that influences the efficiency of cell manipulation^114^. To control the height at which cells are trapped by the DEP cage, proposed strategies include utilizing an inverted quadrupole electrode fabricated at the top of the channel to push cells to the substrate^115^; a double-sided design where two quadrupole electrode patterns are arranged facing each other at the bottom and top of the channel^116^; and a horizontal quadrupole electrode design where two poles are fabricated at the top of the channel and two poles at the bottom (Fig. 6b)^117^. By introducing a dotted-shape grounded electrode at the center of the quadrupole electrode, the electric field gradient can be adjusted through the phase difference of the applied signal to either trap or repel cells from the center of the electrode pattern, further improving the ability to manipulate multipole electrodes with nDEP (Fig. 6c)^118^. The main advantage of using DEP cages for single-cell analysis is that they offer a contact-free approach to trap cells in a region distant from electrodes with minimum influence on cells, thereby facilitating single-cell cultivation^119^, imaging^120^, or electrical characterization^121^. Additionally, as most common buffers used in bioanalyses are of high conductivity, nDEP approaches are advantageous over pDEP approaches due to higher compatibility with on-chip sample processes^122^.

#### Rotating field

**Fig. 7:**
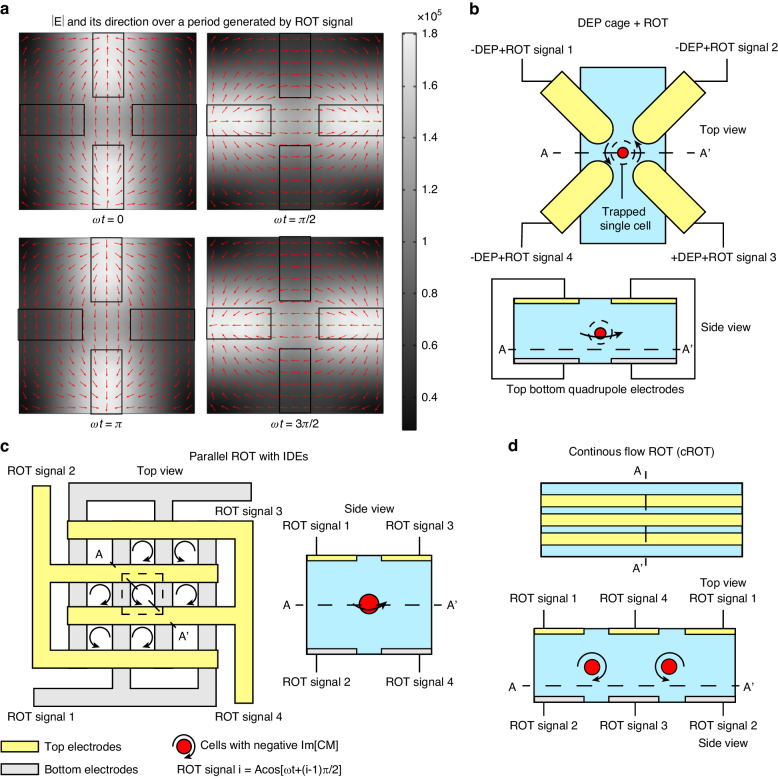
Rotating electric field generated by quadrupole electrodes. **a** Simulations of the rotating electric field over a period. **b** Schematics of single-cell ROT combined with single-cell capture with DEP cage^126^. **c** Parallel single-cell ROT with an array of quadrupole electrodes formed by IDEs^127^. **d** Configuration of electrodes to generate continuous flow ROT perpendicular to the device substrate^128^

The instant intensity distribution of the rotating electric field generated by such electrode configuration over a single period was simulated using the COMSOL finite element analysis tool (Fig. 7a). The rotating polarization density field can induce ROT in the cells at the center. The rotation speed is directly correlated with the dielectric properties of the particles. Therefore, many studies have employed such quadrupole designs to trap single cells with nDEP and extract the dielectric properties at single-cell resolution by visually measuring the rotating speed^123–125^. In this process, single cells are initially trapped by the DEP cage at the center of the quadrupole electrode. Then, the applied DEP signal can be switched to the ROT signal to induce rotation of the cells and facilitate single-cell analysis. One issue is that the DEP cage will disappear when transitioning from the DEP signal to the ROT signal, causing a change in the position of cells and affecting the accuracy of the analysis. This problem can be addressed by directly overlapping the DEP trapping and ROT signals^126^ (Fig. 7b). In addition to the coplanar electrode configuration, electrode poles can also be designed at different layers of microchannel. Quadrupole electrode configurations designed at the top and bottom can be combined with the IDE design to form an array of ROT centers by perpendicularly arranging top and bottom IDEs^127^ (Fig. 7c) or induce continuous-flow electrorotation (cROT) through parallel arrangement of IDE pairs^128^ (Fig. 7d). Both of these designs improve the analysis throughput of ROT and offer a strategy that relieves the issue of multilayer wiring when parallel driving multiple quadrupole electrodes are used.

### Microwell electrodes

**Fig. 8:**
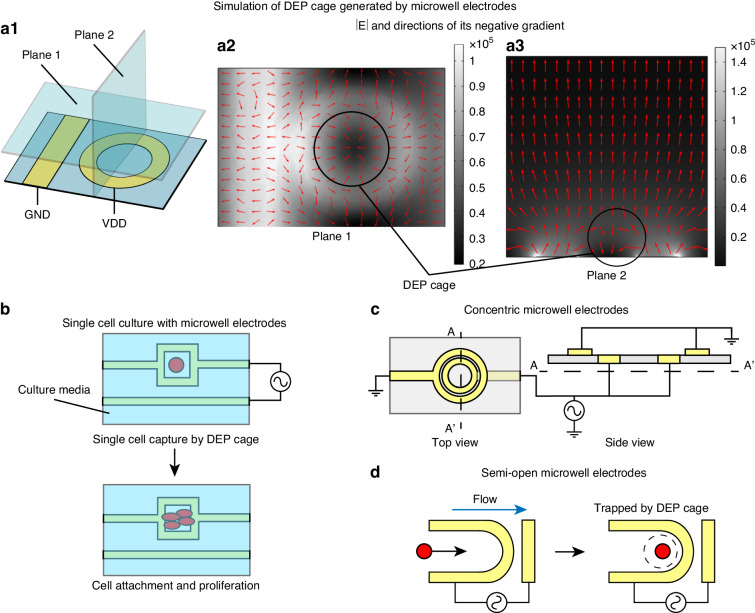
Microwell electrode configurations for DEP cage generations. **a1** Schematic of typical microwell electrodes consisting of a circular-shaped electrode pole. Simulations of the electric field at the horizontal (**a2**) and vertical (**a3**) planes generated by the microwell electrodes. **b** On-chip single-cell culture with microwell electrode^130^. **c** Concentric microwell electrodes^131^. **d** Microwell electrodes with semi-open well to facilitate cell entry^133^

Microwell electrodes are another electrode configuration commonly used to generate a DEP cage. Different from the multipole configuration, where multiple pairs of electrodes surround the minimum electric field region, the microwell design employs only one pair of electrodes with a ring-shaped electrode design to generate the DEP cage (Figure 8a1). We simulated the electric field of a microwell electrode configuration consisting of one ring-shaped electrode and one band-shaped electrode as positive and ground, respectively (Figure 8a2, a3). In this electrode design, the size of the DEP cage was controlled by the open area of the electrode microwell. A larger well can monitor the DEP motion for a group of cells, and a smaller well can trap single cells^129^. With the appropriate cell culture media, the trapped cells can be immobilized and proliferate at the center of the microwell electrode, enabling on-chip single-cell culture^130^ (Fig. 8b). Since nDEP is effective in high conductivity buffer, the DEP cage design relieves the need for buffer substitution between cell capture and culture. In some studies, the microwell electrode pairs were designed as a concentric circle shape, allowing the high electric field region to only appear at the edge of the DEP cage (Fig. 8c). Compared with the multipole electrode design, the structure of the microwell electrode is simpler and thus more scalable to form an array^131^. However, the center of the microwell electrode is completely surrounded by a high electric field region, which may impede cells from effectively entering the DEP cage. To address this issue, the microwell electrode can be designed to have a semi-open shape to allow cells to better access the DEP cage^132,133^ (Fig. 8d). There are also designs that place microwell electrodes at the center of quadrupole electrodes, sequentially achieving the cell accumulation through ACET generated by the quadrupole electrodes and single-cell trapping using DEP cages created by the microwell electrodes^134,135^. Besides, similar to the multipole configuration, the microwell electrode design can also be modified to capture single cells using pDEP. In these designs, one electrode pole is designed in a dotted shape and placed at the center of the electrode microwell to generate the maximum electric field region^136^.

### Microelectrode array (MEA)

Microelectrode arrays can be classified into programmable and non-programmable types according to their operating capabilities. Programmable MEAs offer the advantage of individual addressability for each electrode pixel within the array. This feature enables precise and arbitrary manipulation of single cells, while signals and states of electrodes in non-programmable MEA cannot be individually controlled.

**Fig. 9:**
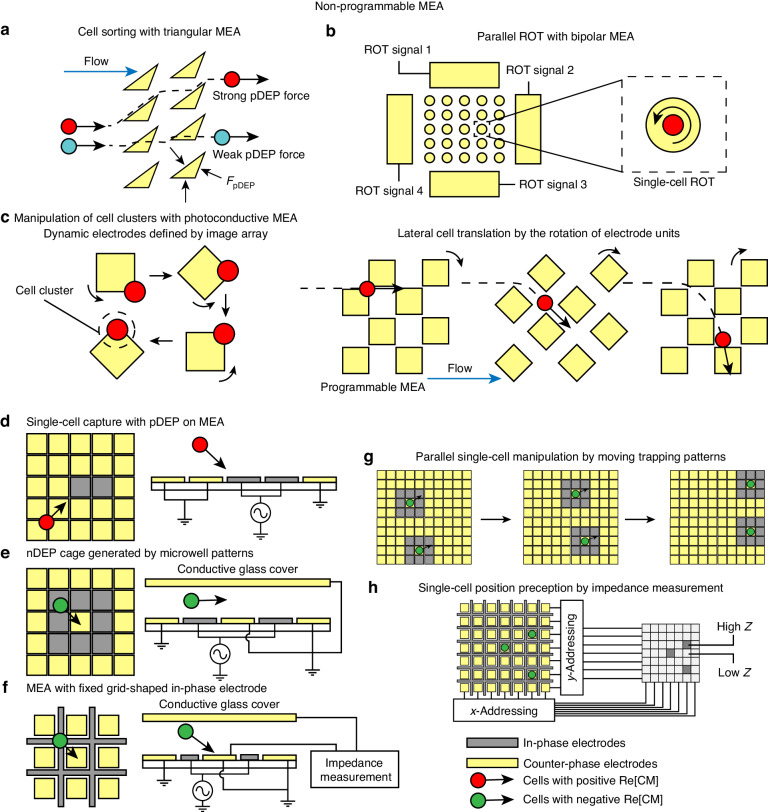
Non-programmable and programmable MEA. Non-programmable MEA (**a–c**): **a** Triangular electrode array for stream-based cell sorting^139^. **b** Parallel ROT with an array of round bipolar electrodes^76^. **c** Single-cell manipulation with photoconductive MEA defined by dynamic projected images^142^. Programmable MEA (**d–h**): **d** Single-cell capture with pDEP^28^. **e** Single-cell capture with DEP cage generated by the microwell pattern^144^. **f** MEA with grid-shaped in-phase electrodes to capture cells with pDEP^145^. **g** Parallel single-cell manipulation by moving the cell trapping patterns^144^. **h** Impedance-based single-cell position perception^145^

Non-programmable MEAs are commonly used to generate an array of universal electric field extremum regions to capture or manipulate single cells in parallel. The shape of a microelectrode unit can be tailored to specific needs, including triangular, rectangular, or other irregular shapes^137,138^. In these cases, non-programmable MEAs function according to similar principles to band electrodes or multipole electrodes. For instance, a triangular MEA can be utilized to separate cells within a continuous flow by employing the DEP force generated at the edge of the electrode coupled with the fluid drag force^139,140^ (Fig. 9a). When MEAs are used for cell trapping, they are capable of generating more widely dispersed electric field extremums than band electrode pairs and provide higher scalability than multipole electrodes. Furthermore, photoconductive electrodes and BPE approaches are more commonly applied in transmitting DEP signals to MEAs than other configurations, as ODEP can empower MEA with more flexibility in single-cell manipulation, and BPE can avoid multilayer wiring during MEA fabrication. An array of BPE driven by a rotating electrode provides a more efficient and simple strategy than an array of multipole electrodes to rotate single cells in parallel^76^ (Fig. 9b). MEA formed by photoconductive electrodes and light patterns allows the precise manipulation of trapped cells by changing the projection of optical patterns^141–143^ (Fig. 9c).

**Fig. 10:**
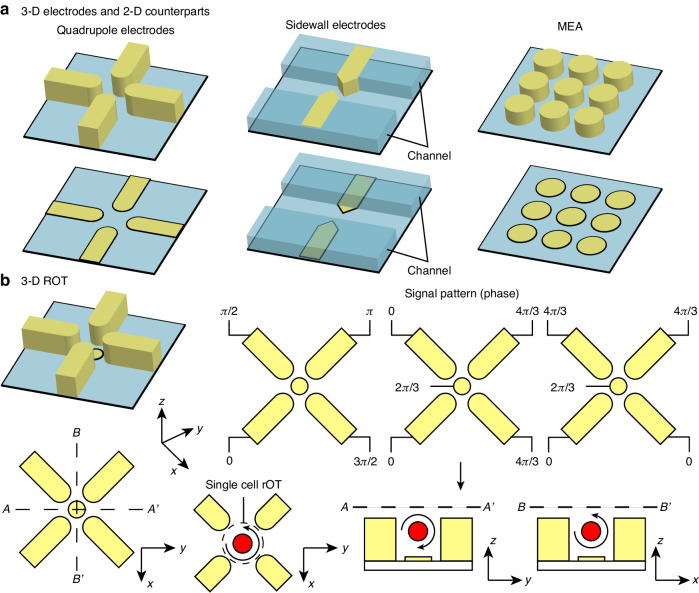
3-D electrodes. **a** Schematic of 3-D electrodes and their 2-D counterparts. **b** Operating schematics of 3-D ROT and the corresponding electrode design^161^

Programmable MEA with individually addressable electrodes possesses a more complex electrical structure but higher capacity in single-cell manipulation than non-programmable MEA. Typical programmable MEA designs feature an array of square micron-sized electrodes to generate an electric field extremum region comparable to cell size to trap single cells at isolated locations. The DEP trapping can rely on either pDEP or nDEP. In the case of pDEP, the high electric field region can be generated by applying certain electrical potential differences to two adjacent electrode pixels^28^ (Fig. 9d). For nDEP, electrode pixels can form a configuration resembling microwell electrodes, where in-phase electrodes surround the counter-phase electrode to create the nDEP cage^144^ (Fig. 9e). Programmable MEA can also be designed to have a fixed grid-shaped in-phase electrode with counter-phase electrode pixels in the grid^145^ (Fig. 9f). The individually addressable electrode pixel enables the manipulation of single trapped cells by digitally shifting the excitation signal configuration between electrode pixels. This feature facilitates the highly parallel software-controlled displacement of cells on programmable MEA along the planned trajectories^144^ (Fig. 10g). Research has also explored the feasibility of employing twDEP to transport single cells on MEA^146^. Some proposed programmable MEA designs incorporate on-chip electrical impedance or optical sensing modules to create a close-loop manipulation-perception system-on-chip (SoC) for single-cell manipulation and sorting (Fig. 10h)^145,147^. Due to the necessity for integration of control and sensing circuits, most of these devices are manufactured following the standard CMOS process^148,149^. A prominent example of a successful commercial DEP-based single-cell manipulation and sorting device is DEPArray, which is based on programmable MEA and a microscope-based optical sensing system^150^. This system has been widely applied to the isolation and single-cell molecular characterization of tumor cells^151–153^. However, to meet the rigorous standards for commercialization in terms of robustness and reproducibility, DEPArray^TM^ does not incorporate an on-chip sensing system, and the microfluidic MEA was designed as a single-use cartridge. While this design redundancy allows for engineering reliability, it positions the DEPArray^TM^ as a benchtop instrument rather than a portable device, which betrays the concept of Lab-on-a-Chip, presenting a challenge that exist for most DEP microfluidic devices.

### 3-D electrode

A 3-D electrode is a vertical extension of a planar electrode, and as such, categorizations applied to planar electrodes are also relevant to their 3-D counterparts (Fig. 10a). A common application of 3-D electrodes is in side-arranged electrode configurations. In contrast to a planar electrode at the bottom of the channel, where the generated electric field will rapidly diminish along the channel height, 3-D electrodes can be extended to a similar height as the channel. This feature allows the electric field to maintain a consistent intensity throughout the height dimension, thus increasing the influential range of the DEP force. Various micro-electro-mechanical systems (MEMS) processes have been employed to fabricate 3-D conductive structures, including techniques such as electroplating^154^, conductive polymer molding^155^, carbonization^156^, and silicon bonding^157^. These 3-D electrodes are often designed to become a part of the channel sidewall directly exposed to the fluid environment^157,158^. An alternative approach to creating 3-D electrodes involves introducing another channel adjacent to the main sample channel, filled with a conductive saline solution or molten solder to enable cDEP^159,160^. Compared to conventional DEP, contactless channel electrodes have advantages like reduced electrochemical effects, minimized Joule heating, and less potential contamination.

3-D electrodes are also employed in multipole electrode configurations to generate DEP cages and induce ROT of single cells^161^ (Fig. 10b). 3-D ROT offers advantages over conventional 2-D ROT as it permits out-of-plane rotation, facilitating 3-D single-cell imaging. The maintained electric field gradient in the vertical direction ensures that ROT responses remain consistent and stable even when cells are positioned at different heights^162^.

3-D electrodes can also be arranged to form an array, where each unit resembles a conductive pillar. In the planar MEA configurations, electrode units (pixels) are closely spaced, and the electrical extremum regions are defined by the patterns of activated electrodes. By contrast, conductive pillars of 3-D configurations are typically distant from each other and collectively function as an array of 3-D multipole electrodes^163,164^. On a macro scale, an open-format device design with a 3-D carbon electrode array has demonstrated the ability to achieve parallel selective single-cell transfers between culture plates^165^. It is worth noting that certain 2-D electrode configurations, such as band electrode pairs, do not have 3-D counterparts. Elevating their electrode height would obstruct the fluid flow and introduce dead volume into the fluid channel, thereby having no impact on the effective range of DEP.

### Summary of discussion

In the design process, the parameters of the DEP signal, such as amplitude and frequency, are modifiable during experiments. Therefore, the key factor that needs to be determined is the electrode pattern, which should be considered in conjunction with the required DEP effect. In the dynamic wave field, the phases of applied signals should be predetermined along with the electrode patterns to induce the traveling wave or rotational field, whereas the standing field only concerns physical geometry.

To provide readers with a clear understanding and reference, the relationship between the introduced electrode patterns and their induced DEP effect is summarized in Table 2. Note that in this table, we primarily focus on the corresponding DEP effects employed in the cellular analysis. For instance, the microwell electrode, or theoretically any other electrode patterns, can induce the ACET flow, but there are few corresponding applications for cell manipulation or cellular analysis.

**Table 2:** Electrode patterns and induced DEP effects for cellular analysis

Electrode Pattern	2D/3D	Induced DEP Effects For Cellular Analysis
Cascaded Band Electrodes	2D	Standing Wave DEP, Traveling Wave DEP, L-DEP, ACET
Quadrupole Electrodes	2D	Standing Wave DEP, Electrorotation, ACET
	3D	Standing Wave DEP, Electrorotation
Microwell Electrodes	2D	Standing Wave DEP
Micro Electrode Array	2D	Standing Wave DEP, Electrorotation, Traveling Wave DEP
	3D	Standing Wave DEP, Electrorotation

## Physical design

In addition to electrodes, physical designs, such as the channel or other facilitating components, play a critical role in DEP microfluidic devices. This section will provide an overview of common physical designs, including microwells, channel-based microtraps, convex micropillars, microvalves, and droplet generation structures.

### Microwells and channel-based microtraps

On-chip single-cell analysis normally requires individual cells immobilized on the device for prolonged biochemical reactions. However, the introduction of additional reactants can alter the dielectric properties of the fluid buffer, affecting the corresponding DEP force. One approach to address this issue involves the integration of passive micro-trapping structures on DEP microfluidic devices to physically immobilize and encapsulate single cells that are initially captured by DEP force. Similar to the well plate that allows parallel storage and processing of multiple biosamples, a microwell array at the bottom of the channel provides a straightforward approach to parallel immobilization of single cells within the channel. These microwells create dead volumes to prevent trapped cells from being carried away by the fluid flow. Alternatively, the planar trapping structure can also be designed on one side of the channel. Electrodes at the bottom can direct cells into microtraps at the side of the channel with DEP force for subsequent analysis.

#### Microwells

**Fig. 11:**
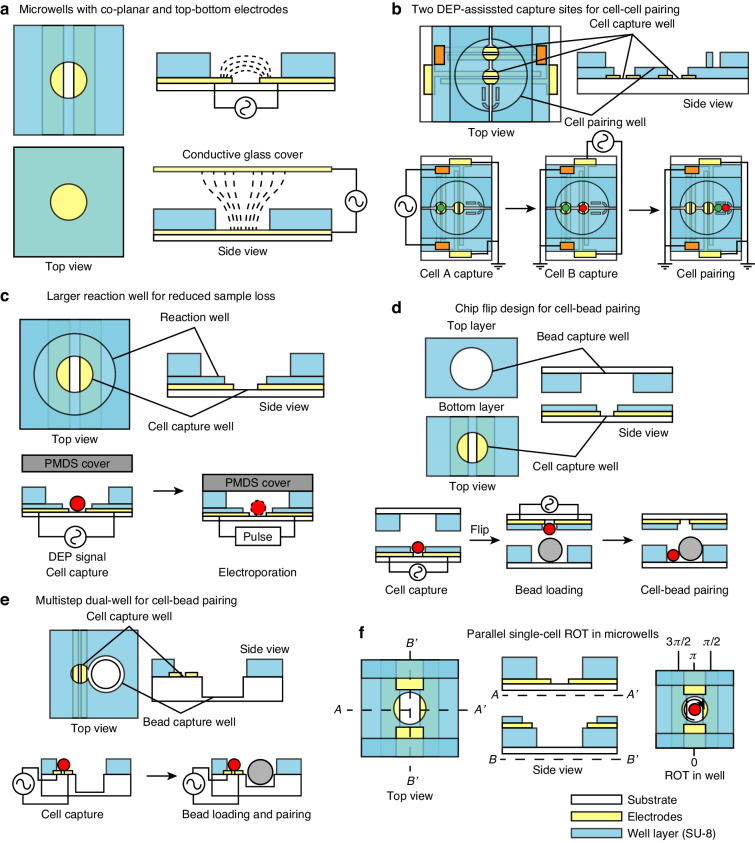
Schematics of different DEP-assisted microwell designs for single-cell analysis. **a** Micowell designs with coplanar and top-bottom electrodes for DEP generation. **b** Microwell design with two capture sites for cell pairing^174^. **c** Microwell design with a larger reaction well for reduced intracellular loss after electroporation. Redrawn from^177^. **d** Dual-microwell design for cell-bead pairing by chip flip^179^. **e** Coplanar dual-microwell design for cell-bead pairing^178^. **f** Microwell design with quadrupole electrodes for parallel ROT in wells^180^

The gravitational force of cells is nearly negligible at the micron scale. Consequently, simple microwell structures alone are normally insufficient for efficiently capturing and trapping cells in microfluidic devices. To address this limitation, DEP force was introduced to facilitate active single-cell capture from a fluid environment. Microwells can be fabricated by patterning a permanent photosensitive epoxy at the top of the planar band electrode pairs. In this configuration, a pair of electrode edges are exposed at the bottom of the microwell to generate the DEP force from the bottom^166^. The insulative microwell layer shields the electric field from unexposed parts of the electrodes. As the trapped single cells are in close proximity to both electrode poles, the induced change in impedance can reflect the number of trapped cells and facilitate the impedance-based single-cell characterization^167^. Furthermore, the close contact between cells and both electrode poles allows electroporation to lyse cells in microwells by applying a high electrical potential difference, which is essential for certain intracellular material analyses that necessitate the disruption of cell membranes^168^. Another configuration involves direct patterning of the microwell structure at the opposing conductive layer at the channel top and bottom. Here, the microwell layer not only functions as the physical cell trapping structure but also defines the shape of the exposing electrode at the channel bottom to generate a non-uniform electric field^169,170^ (Fig. 11a). Microwells can also form isolated reaction chambers for each individual cell by sealing the well openings. In a closed-channel device, sealing can be achieved by applying a flexible PDMS channel cover with mechanical force or introducing a layer of oil^171^. In an open-format device without a channel cover, the microwell array can be sealed by placing a semi-permeable membrane or a glass slide over it^172,173^.

The shape and arrangement of microwells can be further modified to accommodate specific single-cell applications. For example, a microwell unit can be modified to have two DEP single sources and capture sites to trap two different cell types in each microwell, followed by subsequent cell pairing^174,175^ (Fig. 11b). Studies have also demonstrated that two cells can be trapped in one microwell by increasing the well depth^176^. To minimize the loss of intracellular materials in the microwell after cell lysis, a smaller cell-capturing well can be placed within a larger reaction well^177^ (Fig. 11c). In some advanced single-cell analyses requiring pairing between single-cells and functionalized microbeads, a larger bead-trapping well can be incorporated alongside or opposing the smaller cell-trapping well, forming a dual-well structure^178,179^ (Fig. 11d, e). Additionally, multipole electrodes can be integrated into the microwell structure to facilitate parallel ROT followed by single-cell trapping^180^ (Fig. 11f).

#### Channel-based microtraps

**Fig. 12:**
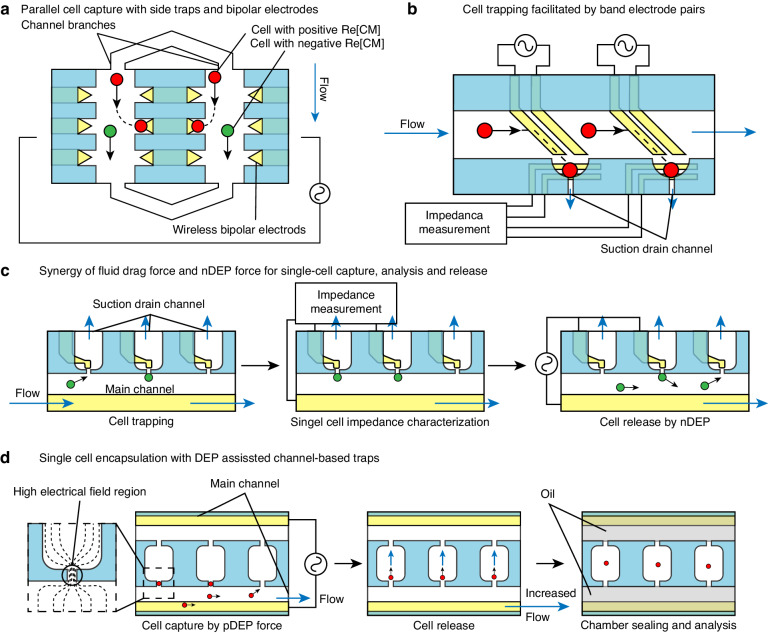
Operating schematics of DEP-assisted channel-based traps. **a** Parallel selective single-cell capture with side traps and an array of BPE^75^. **b** Single-cell capture with side traps and single-cell impedance characterization assisted by tilted electrode pairs^181^. **c** The synergy of fluid drag force and nDEP force for multiplexed single-cell capture, analysis, and release^182^. **d** DEP-assisted single-cell encapsulation in channel-based traps^183^

Apart from microwells positioned at the channel bottom, 2-D trapping chambers are another type of fixed physical design that can also create dead volume within the channel for cell trapping. These 2-D structures are typically patterned alongside the channel and are located at the side of the channel. Electrodes can be integrated into these microstructures to laterally attract cells into the chamber. Since the DEP force diminishes exponentially from the electrode pairs, designing electrodes at the side of the channel requires a limited channel width to ensure cells can be drawn into chambers. For sufficient throughput, more branched channels can be integrated into a single device^75,77^ (Fig. 12a). Some designs also incorporate band electrode pairs to facilitate the lateral movement of cells toward traps at the side of the channel^181^ (Fig. 12b). In certain designs, drain channels are integrated at the lateral bottom of these side traps. These drain channels become blocked when a single cell is trapped, enabling synergy of fluid drag force and DEP force for cell capture and release^181,182^ (Fig. 12c).

Design variations can allow channel-based traps to serve as isolated microchambers as well as function as a physical boundary to constrain the distribution of the electric field. These designs typically include narrow and short channel braches with microchambers alongside the main channel path. Electrode pairs are arranged at two ends of narrow branched channels. The generated electric field intensifies as field lines pass through these narrow channels, inducing DEP force^183,184^ (Fig. 12d). This type of microfluidic design, which uses insulator structures rather than electrodes to produce non-uniformity in the electric field to induce DEP, is referred to as insulator-DEP (iDEP)^185^. Detailed physical principles and more modified iDEP channel structures have been extensively reviewed^186^. One of the most evident advantages of iDEP is that samples will not come into direct contact with the electrode, similar to cDEP, relieving the influence of Jule heating and electrical chemical contamination. Like microwell structures, channel-based traps can also be sealed to compartmentalize cells into micro-reaction chambers by introducing an immiscible phase, such as oil, into the main channel^183^.

### Convex microstructures

**Fig. 13:**
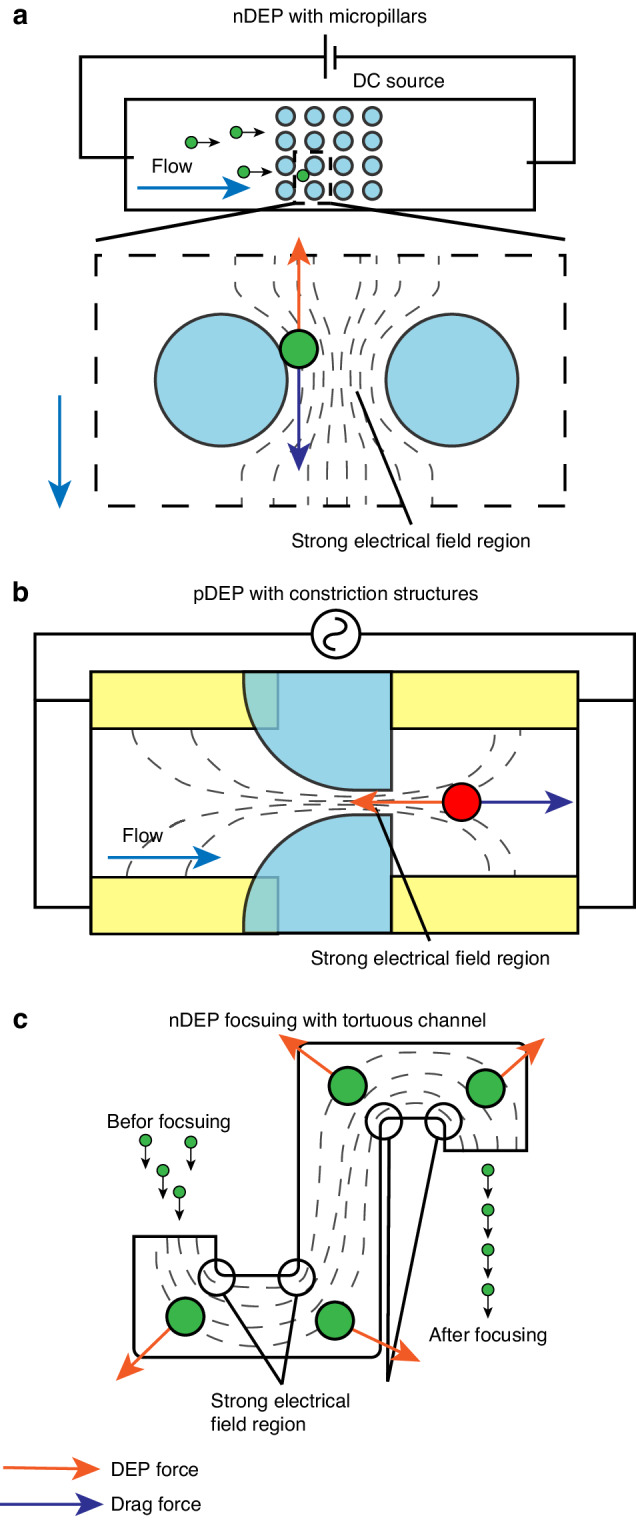
Non-uniform electric field constrained by convex microstructures. **a** Micropillars for cell capture with nDEP force^209^. **b** pDEP force induced by constriction structures for cell capture with pDEP^191^. **c** Cell focusing with nDEP from the electric field constrained by tortuous channel^190^

Convex microstructures can be utilized in microfluidic devices to induce a non-uniform electric field in an electrodeless approach. The operating principle of convex microstructures is essentially the same as the previously described microchambers connected with narrow channels. Both designs fall under the category of iDEP and utilize the narrow gaps formed by insulating structures to interfere with DC electric field lines, resulting in the formation of field extremum regions. However, in contrast to channel-based traps, such convex structures do not create dead volumes or chambers in the channel to encapsulate cells. Convex microstructures are also independent of the channel and can form array patterns for higher parallelization and throughput. For instance, when a DC electric field penetrates an array of micropillars arranged in a fluid channel, the electric field intensifies in the area between two pillars due to the higher conductivity of the liquid media compared to the insulator structures^187–189^ (Fig. 13a). By applying the electric field parallel to the flow direction, the nDEP force that opposes the flow drag force is induced, which can be employed to trap cells in the channel. The geometry and arrangement of micropillars can be further modified to capture single cells^187^. As with other DEP-based cell capture techniques, selectivity can be achieved by modulating the proper frequency to manipulate the DEP states of different cells. Convex obstacle structures can also be designed as a part of the channel, such as constriction structures and serpentine or tortuous channels^190,191^ (Fig. 13b, c). Serpentine channel designs are normally employed to induce a non-uniform electric field perpendicular to the flow direction, enabling stream-based particle focusing or separation using DEP force^190,192^. Nevertheless, compared to micropillar arrays, these designs cannot form an array and thus are typically used in scenarios where lower throughput is sufficient.

### Microvalves

Microvalve structures are another microfluidic approach for creating on-chip micro-reaction chambers. These physical designs use the multilayer flexible PDMS channels. The basic valve structure consists of a top control channel made with a highly crosslinked PDMS control stacked on a relatively weakly crosslinked PDMS flow channel at the bottom. When pressure is applied to the control channel, the intersection area of the bottom flow channel is deformed, allowing for control of the flow rate based on the applied pressure. With sufficient pressure, the deformation can completely block the flow channel. Consequently, two microvalve structures located at the ends of a section of the channel can isolate a chamber for single-cell analysis. These valve structures can also divide the channel into multiple segments for multi-step biochemical reactions, such as gene library preparation^193^. However, in contrast to microwells, microvalve units are more complex in their fabrication and consume a larger area, which can have a negative impact on the system engineering reliability.

**Fig. 14:**
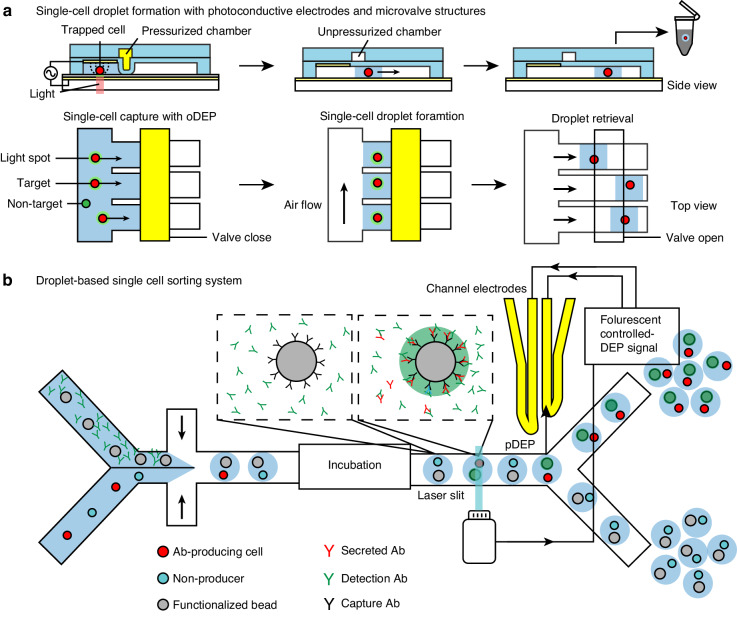
DEP-assisted microvalve and droplet microfluidic devices for single-cell analysis. **a** A microvalve device for single-cell droplet formation with DEP force generated by photoconductive electrodes^195^. **b** A droplet microfluidic device employing nDEP-controlled droplet manipulation for single-cell sorting^199^

With the assistance of DEP force, single cells can be immobilized at predefined sites within the channel. Subsequently, the valves can be closed to compartmentalize individual cells for subsequent analysis^194,195^. When single cells are immobilized in the traps, air can be introduced to remove liquid buffer from the main channel. The valves can then close the main flow channel output and open the drain channel output. This will allow liquid medium in traps to carry cells out of the device, forming single-cell droplets for off-chip analysis^195^ (Fig. 14a). Different from the previously mentioned structures, which interact with the electric field to induce spatially constrained DEP force, the operation of microvalves and DEP are relatively independent of each other, solely serving as a physical structure to compartmentalize single cells.

### Droplet microfluidics

Droplet microfluidic devices utilize dynamically generated water-in-oil droplets as a micro-reaction chamber to compartmentalize single cells. Droplet generation is typically a passive process achieved by introducing one immiscible fluid into another. Multiple external techniques, such as electrical, magnetic, and mechanical approaches, have been employed to facilitate this process. Physical principles, techniques, and hydrodynamic optimization of droplet microfluidic devices have been extensively reviewed^196,197^. In contrast to microwell and microvalve devices, where cell encapsulation is performed in parallel with the assistance of fixed physical structures, droplets are formed sequentially without requiring physical structures. This characteristic allows droplet devices to achieve a higher sample throughput. However, since single cells and other necessary reactants are encapsulated sequentially, such devices are unsuitable for multi-step biochemical reactions. By comparison, microvavle and microwell systems are both compatible with multi-step reactions^172,198^.

In droplet microfluidics devices, DEP force plays a significant role in manipulating water-in-oil droplets. As the aqueous droplets are more polarizable than the carrier oil, pDEP is dominant under the effect of the external electric field. By encapsulating a fluorescent antibody with a capture antibody-coated microbead together with single cells into a droplet, cells secreting target antibodies will accumulate fluorescent levels on the co-encapsulated beads during incubation. The microscope detects fluorescent signals from passing droplets and controls the on and off of the DEP signal. When droplets containing target cells pass the detection lens, electrodes will be activated with a DEP pulse that attracts the droplet into the collection channel. Since the collection channel has a higher hydro resistance than the waste channel, droplets below the fluorescent threshold spontaneously flow to the waste channel. This forms the architecture of a single-cell sorting system using droplet microfluidics^199^ (Fig. 14b). The most common electrode configuration used to control droplets is the 3-D channel electrode arranged at a side of the main sample channel, as it can generate a more uniform and stronger DEP force in the narrow channel design.

### Summary of discussion

In this section, we introduced typical physical designs that operate together with DEP force in the cellular analysis. The functions of these physical designs can be summarized in these two aspects. First, these physical structures geometrically constrain the boundary conditions of physics, directly affecting the distribution of the induced electric field. Second, in single-cell analysis, physical designs are able to create isolated microchambers for individual cells, which is essential for multiple single-cell biochemical reactions to prevent cross-contamination. The DEP force plays a crucial role in encapsulating single cells into reaction chambers and in controlling the formed reaction chambers (*i.e*., droplets). A systematic comparison of these physical designs is provided in Table 3 to offer readers a straightforward understanding of the content.

**Table 3:** Comparison of different DEP-assisted microstructures

Structures	Physical Functions	Structure Designs	Applications	Throughput or Sample Volume
Microtraps	Fixed physical chambers and constraints of the electric field	Microwells	Micro-reaction Chambers^177^ Singe-cell capture^166,180^ Cell-cell pairing^180,181^ Cell-bead pairing^178,179^	10^2^ ~ 10^4^ wells
		Channel-based Microtraps	Single-cell capture^77,182^ Micro-reaction chambers^183^	10 ~ 10^2^ traps
Convex Microstructures	Constraints of the electric field	Micropillars	Cell capture^159^	<10^2^ pillars
		Channel	Cell capture^187^	<10 constriction
		Tortuous Channel	Cell focusing^190^	<10^2 ^μl/hr
Microvalves	Fixed physical chambers	Microvalves	Micro-reaction chamber^194^ Single-cell droplet formation^195^	10 ~ 10^2^ valves
Droplet Microfluidics	Dynamic physical chambers that can be manipulated by DEP	Droplets	Single-cell droplet formation^208^ Cell-bead pairing^199^	~10^2 ^μl/hr ~10^3^ droplets/s

## Conclusion and outlook

This review provides an in-depth analysis of the role of DEP in single-cell microfluidic systems, with a focus on its physical principles and the engineering designs of DEP operating structures. DEP offers a powerful label-free approach to manipulate dielectric particles with selectivity. From the perspective of particle manipulation, most microfluidic devices leverage specifically designed electrodes and physical structures to manipulate particles in a fixed pattern, such as focusing, trapping, and rotating. While there are many complex manipulation patterns for specific applications, commercialized platforms primarily utilize DEP to capture and aggregate cells or particles from suspensions, such as the Passonic bacteria counter^200^ and Shimadzu IG-1000 Plus nanoparticle analyzer^201^. In contrast, programmable electrode arrays, such as the DEPArray™, enable parallel manipulation of cells along arbitrary routes, facilitating high-precision single-cell sorting and characterization using DEP combined with immunofluorescence labeling techniques.

From the perspective of selectivity, the potential application for DEP is limited as DEP response is based on the physical properties of particles, such as conductivity, permittivity, and dimension. On the one hand, these properties may have a large variation within the same type of cells, which increases the probability that different types of cells could exhibit similar physical properties. On the other hand, the dimension of data reflected by DEP is quite limited. The parameter fitting process used to estimate these physical properties of cells is typically based on a simplified multishell model. Therefore, commercialized microfluidic platforms have focused on applications where a relatively large difference in physical properties is observed between target and non-target cells, such as tumor cells in the blood.

Moving forward, the greatest promise for DEP lies in integrating its manipulation capability with other specific labeling methods to enhance selectivity. However, this poses challenges in terms of additional detection systems and their synergy with DEP. For instance, combining DEP with immunofluorescent labeling requires multichannel fluorescent microscopy for closed-loop manipulation and perception. As DEP technologies mature in single-cell applications, the key challenge will be to efficiently integrate it with other detection methods, necessitating further integration of detection systems with DEP systems and higher parallelization in sample processing. Future research efforts should focus on developing more efficient and integrated platforms that seamlessly combine DEP manipulation with high-specificity detection methods, ultimately advancing the microfluidic single-cell analysis systems.
